# The Contribution of Ethnicity to the Association of *MTHFR* Variants C677T and A1298C with Autism Spectrum Disorder: A Meta-Analysis

**DOI:** 10.3390/brainsci16010093

**Published:** 2026-01-16

**Authors:** Yining Pan, Brooklyn McDill, Marie Mooney

**Affiliations:** 1 Department of Public Health, Brooks College of Health, University of North Florida, Jacksonville, FL 32224, USA; yining.pan.1@student.unimelb.edu.au; 2 Department of Medical Biology, Faculty of Medicine, Dentistry and Health Sciences, University of Melbourne, Melbourne, VIC 3010, Australia; 3 The Walter and Eliza Hall Institute of Medical Research, Melbourne, VIC 3052, Australia; 4 School of Nursing, Brooks College of Health, University of North Florida, Jacksonville, FL 32224, USA; 5 Department of Biology, College of Arts and Sciences, University of North Florida, Jacksonville, FL 32224, USA

**Keywords:** ASD, *MTHFR C677T*, *MTHFR A1298C*, Egyptian, meta-analysis

## Abstract

**Background:** Common polymorphisms in the *MTHFR* gene, *C677T* and *A1298C*, have been associated with increased risk for psychiatric neurodevelopmental disorders, including autism spectrum disorder (ASD). However, studies provide conflicting evidence for the strength of the association with ASD based on both the allelic variant and population structure of the cohorts studied. **Methods:** Using systematic literature search and selection criteria, we calculated ASD-associated odds ratios for the two most-reported MTHFR variants. Twenty-two articles reported the association between *MTHFR C677T* and ASD, including 13913 subjects (4391 cases, 9522 controls). Nine articles, including 3009 subjects (1462 cases, 1547 controls), evaluated the link between *MTHFR A1298C* and ASD susceptibility. **Results:** We identified a statistical association between ASD and the *MTHFR C677T* variant, regardless of race or ethnicity. However, there was no statistical support for an association between ASD and the *MTHFR A1298C* variant. In both cases, substantial-to-considerable residual heterogeneity remained (I^2^ ~67% and 73%, respectively). Exploring the heterogeneity by meta-regression on race/ethnicity, the African (Egyptian) cohort with *MTHFR C677T* variants had a higher ASD susceptibility than Asian or European cohorts in most models, though this susceptibility difference was not observed between Africans and Europeans for the homozygous case (TT vs. CC). Similarly, the African (Egyptian) cohort with *MTHFR A1298C* variants also had a higher ASD susceptibility than Asian or European cohorts in most models, though this susceptibility difference was not observed between Africans and Asians for the homozygous case (CC vs. AA). **Conclusions:** Our findings support previous analyses that identified a statistical association between ASD and the *MTHFR C677T* variant but none between ASD and the *MTHFR A1298C* variant. We also reveal a greater potential for these variants to exacerbate ASD phenotypes in an African (Egyptian) cohort. Future studies should assess the mechanistic contribution of these variants to MTHFR function, especially potential hypomorphic sensitivity in individuals with African (Egyptian) ancestry.

## 1. Introduction

**Figure 1:**
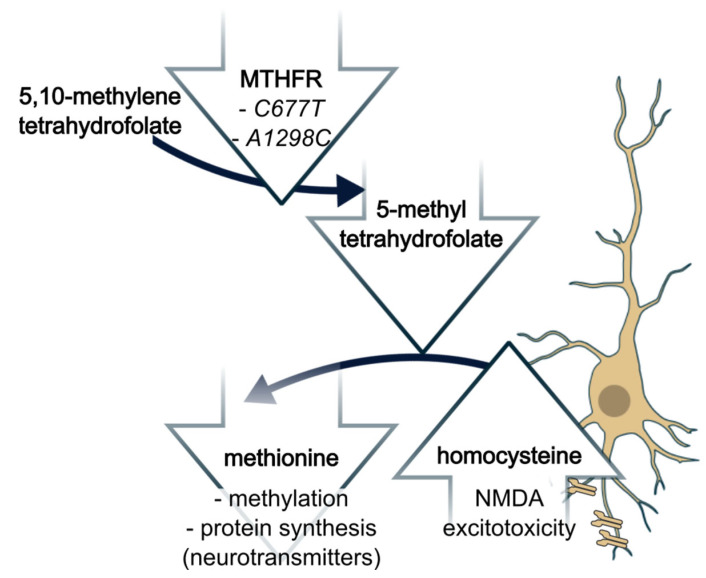
Functional role of MTHFR in folate metabolism and conversion of homocysteine to methionine. Hypomorphic variants reduce the availability of the methyl donor for the conversion, 5-methyltetrahydrofolate, resulting in homocysteine accumulation. Neurons are sensitive to both homocysteine accumulation and reduced methionine. Homocysteine accumulation can lead to excitotoxicity via NMDA receptors, and reduced methionine has wide-ranging effects, including suppression of neurotransmitter synthesis. Illustration created from NIAID NIH BioArt Source, images 23, 31, 424 https://bioart.niaid.nih.gov/ (accessed on 11 June 2025) in Inkscape v1.4.2.

Autism is a neuropsychiatric disorder that emerges during the first three years of life. Individuals with ASD often experience challenges in social communication and interaction, display excessive repetitive behavior, and have restricted interests. The disorder presents as an array of symptoms with varying severities; thus, autism is a spectrum disorder (ASD). Genetic and environmental factors contribute to this phenotypic spectrum. Genetic evidence includes a high concordance rate, exceeding 60%, among monozygotic twins [1,2]. More specifically, hypomorphic variants in the 5,10-methylenetetrahydrofolate reductase (MTHFR) gene reduce folate metabolism and potentially contribute to the neurodevelopmental phenotypes [3,4]. One known physiological effect of hypomorphic MTHFR activity is a depletion in the available pool of the MTHFR metabolite methyltetrahydrofolate. Methyltetrahydrofolate is the primary methyl donor used for transmethylation of homocysteine, a neurotoxic compound, to methionine, an essential amino acid involved in neurotransmitter synthesis (Figure 1). Despite accumulating evidence of MTHFR hypomorphism contributing to psychiatric disorders, few studies have collected consistent genotypic, physiologic, and diagnostic measures [5]. Most often, only genotype–phenotype relationships are available; large-scale studies and meta-analyses associating *MTHFR* variants with ASD have reached inconclusive or contradictory results. Here, we examine the two most frequent variants, *C667T* and *A1298C*, and their role in ASD with respect to background genetic architectures influencing the strength of these variants as phenotypic modifiers. Overall, more severe forms of ASD are thought to correlate with greater reductions in enzyme activity; the *C667T* allele reduces enzyme activity up to 70%, whereas the *A1298C* allele reduces activity by ~40% [6]. The *A1298C* allele results in a less hypomorphic enzyme; however, when observed as a compound heterozygote with the *C677T* allele, the overall effect phenocopies the homozygous *C677T* [7].

Studies have investigated the allele frequencies of the *C677T* and *A1298C* mutants of the *MTHFR* gene in autistic patients compared to healthy controls, but the results are conflicting. For example, James et al. [8] revealed a significant association of both *C677T* and *A1298C* variants with ASD, as did El-Baz et al. [9], although in the latter case, the combined contributions revealed significance, while the individual alleles did not. However, neither Sener et al. [10] nor Zheng et al. [11] found statistically significant frequency differences in the *MTHFR C677T* allele presence between autistic and nonautistic children; they did not assess *A1298C*. Conversely, Meguid et al. reported an increased ASD risk of *A1298C*, but not *C677T*, in an Egyptian cohort [12]. These studies were conducted across different geographical regions (and ethnicities), limiting their global generalization: Africa (Egyptian) [9], Asia (Han) [11], Europe (White American) [8], and the Middle East (Turkish) [10]. The limitations in these studies extend to ours; we include an ethnic designation in parentheses when it is consistent within the cohort.

Several recent meta-analyses (within the last five years) have returned inconclusive findings on the association between the two *MTHFR* variants and ASD. One analysis, including fifteen studies, concluded that the *MTHFR C677T* variant, but not the *A1298C* variant, is a significant risk factor for ASD [13]. This study population was Caucasian and Asian dominated. Another recent meta-analysis included seventeen studies and revealed a significant association of both *MTHFR C677T* and *A1298C* with ASD [14]. Other studies stratified the risk by race/ethnicity but reported different findings. Fang et al. found that *C677T* was associated with a higher risk of ASD in Caucasian and Asian populations, while in subgroup analysis, *A1298C* was a risk factor for ASD in African populations [15]. Conversely, Sadeghiyeh et al. found *C677T* was a risk factor across Caucasian, Asian, and African cohorts, and recessive *A1298C* increased the risk of ASD only in Caucasians [16].

A comprehensive meta-analysis will consolidate the conclusions from these studies regarding the association of *MTHFR* variants with the risk of ASD. Identifying susceptible populations could be useful for planning and implementing public health and medical prevention and treatment programs. Therefore, we conducted a meta-analysis, including all eligible published observational studies, to investigate the association of *MTHFR C677T* and *A1298C* with ASD and stratified the association by race/ethnicity.

## 2. Materials and Methods

The protocol is retrospectively registered with the Open Science Framework. Project ID 3q2jc and registration ID ju2ma.

### 2.1. Literature Review and Selection

The study followed PRISMA guidelines [17]. Literature review of publications between 1 January 1980 (first inclusion of autism diagnosis in the Diagnostic and Statistical Manual of Mental Disorders, Third Edition, DSM-III [18]) and 23 May 2024. The literature came from PubMed, Medline, Google Scholar, and PsycINFO databases. The search terms were ((‘MTHFR’) OR (‘methylenetetrahydrofolate reductase’) OR (‘677C-T’) OR (‘C677T’) OR (‘677C>T’) OR (‘1298CC’) OR (‘1298A>T’) OR (‘1298AC’) OR (‘A1298C’)) AND ((‘Autism’) OR (‘ASD’) OR (‘Autism Spectrum Disorders’)). No restrictions were added; search parameters were set as default, including text availability and article type in PubMed and Medline, article type in Google Scholar, and search limits in PsycINFO.

Eligible studies met the following criteria: (i) published in peer-reviewed journals in the English language; (ii) original observational research in human subjects; and (iii) reported or had sufficient information to calculate the odds ratio (OR) of ASD among *MTHFR C677T* or *A1698C* carriers compared to non-carriers. The first author (Y.P.) screened initial search results based on title and abstract. Potential studies were assessed in full text and included if they satisfied the selection criteria. A second reviewer (M.M.) verified search results. All the authors (Y.P., B.M., and M.M.) reached a final consensus on the eligibility of studies. Additional details in Appendix A.

### 2.2. Study Appraisal and Risk of Bias in the Individual Studies

The Newcastle–Ottawa Scale was used to appraise the quality of the included studies [19]. The scale consists of a maximum of 10 stars for case–control studies. It assesses a study in three categories: the selection of the study groups (maximum of 4 stars), the comparability of the groups (maximum of 2 stars), and the ascertainment of the exposure of interest (maximum of 4 stars). For comparability, we assessed whether the case and control groups were age- and/or sex-matched. We considered studies rated 6–10 stars as high quality and those rated 0–5 stars as low quality. Two reviewers (Y.P. and B.M.) conducted the assessment independently. Disagreements were discussed, and M.M. acted as a tie-breaking evaluator.

Funnel plots and Egger’s regression tests were used to formally assess the risk of publication bias [20]. If Egger’s regression test showed significant asymmetry in the funnel plots, we used the trim-and-fill method to compute the pooled ORs corrected for small-study effects [21,22].

### 2.3. Data Extraction

Data on the following variables were extracted: (i) name of the authors; (ii) year of publication; (iii) name of the journal; (iv) study design (prospective, retrospective, or cross-sectional); (v) *MTHFR* variants being studied; (vi) study location (Africa, Asia, or Europe); (vii) diagnostic tool (DSM-IV, DSM-V, or others); (viii) setting of study (hospital-based or population-based); (ix) sample matching criteria (age-matched, sex-matched, or others); (x) number of ASD cases; (xi) number of controls; and (xiii) number of each genotype of *MTHFR C677T* or *A1298C*.

### 2.4. Statistical Analysis

We calculated the ORs of ASD among *MTHFR C677T* or *A1298C* homozygous individuals compared to two control cohorts: (1) individuals homozygous for the reference allele, or (2) the homozygous reference cohort also including heterozygous individuals. Other models are also available (Supplementary Materials). The ORs were pooled using a random effect model. The weight of each study was determined by the inverse variance method. We calculated the heterogeneity variance tau squared with a restricted maximum likelihood estimator [23]. We employed Knapp–Hartung adjustments to calculate the confidence interval around the pooled ORs [24]. The Cochrane Q and I^2^ statistics measured heterogeneity. Leave-one-out sensitivity analysis was performed using the metainf function in the meta package by sequentially omitting one study at a time.

We also conducted univariate meta-regression to identify race/ethnicity differences in the association of *MTHFR C677T* and *A1298C* with ASD. We approximated the study sample’s race/ethnicity using the continent where the study was originally conducted. Furthermore, we conducted multiple meta-regressions to adjust for potential study-level covariates, including publication year, diagnostic tool (DSM-IV, DSM-V, or others), and study quality, assessed as the total Newcastle–Ottawa Scale Star score. Covariates included in the final meta-regression models were selected based on the proportion of between-study heterogeneity explained (pseudo-R^2^), with preference given to models yielding the highest pseudo-R^2^.

All statistical analyses conducted in R version 4.3.3 used the metafor package to generate pooled ORs and forest plots [25] and the meta package for sensitivity analyses and meta-regressions [26]. Statistical significance was set to *p*-values < 0.05.

## 3. Results

**Figure 2:**
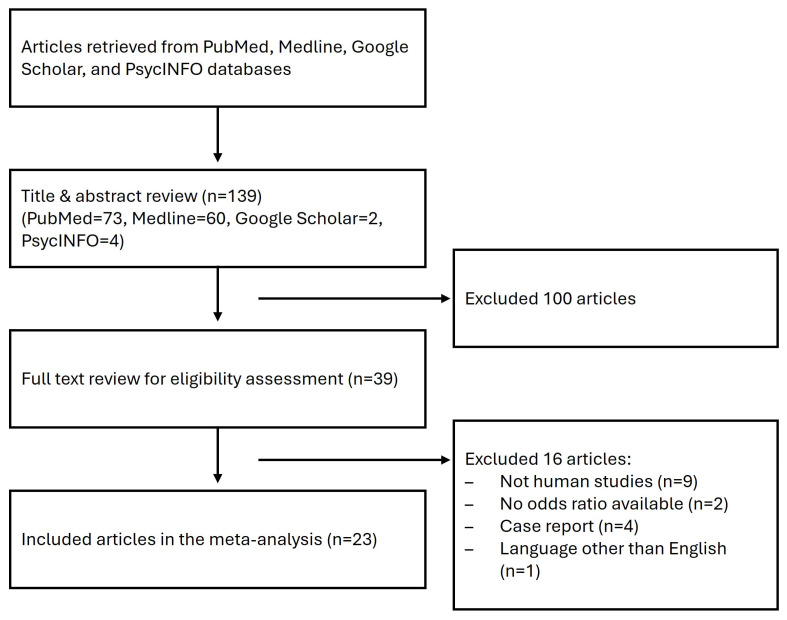
Flowchart of the meta-analysis inclusion and exclusion criteria.

After screening, 23 out of 139 citations were included in the meta-analysis (Figure 2) [8,9,10,11,12,27,28,29,30,31,32,33,34,35,36,37,38,39,40,41,42,43,44]. Of note, one article conducted two separate investigations in simplex and multiplex families [36]. Four articles were conducted in Africa [9,12,34,43], thirteen in Asia [11,27,28,30,31,33,35,37,38,39,40,42,44], and six in Europe [8,10,29,32,36,41]. In particular, all the included studies conducted in Africa were specifically from Egypt. All the included studies were considered to be of high quality except for one study [29], which did not specify the selection and definition of controls, nor did it match the groups by age or sex (Supplementary Table S1). Three disagreements in selection and scoring were discussed and resolved by consensus without tie-breaking.

**Figure 3:**
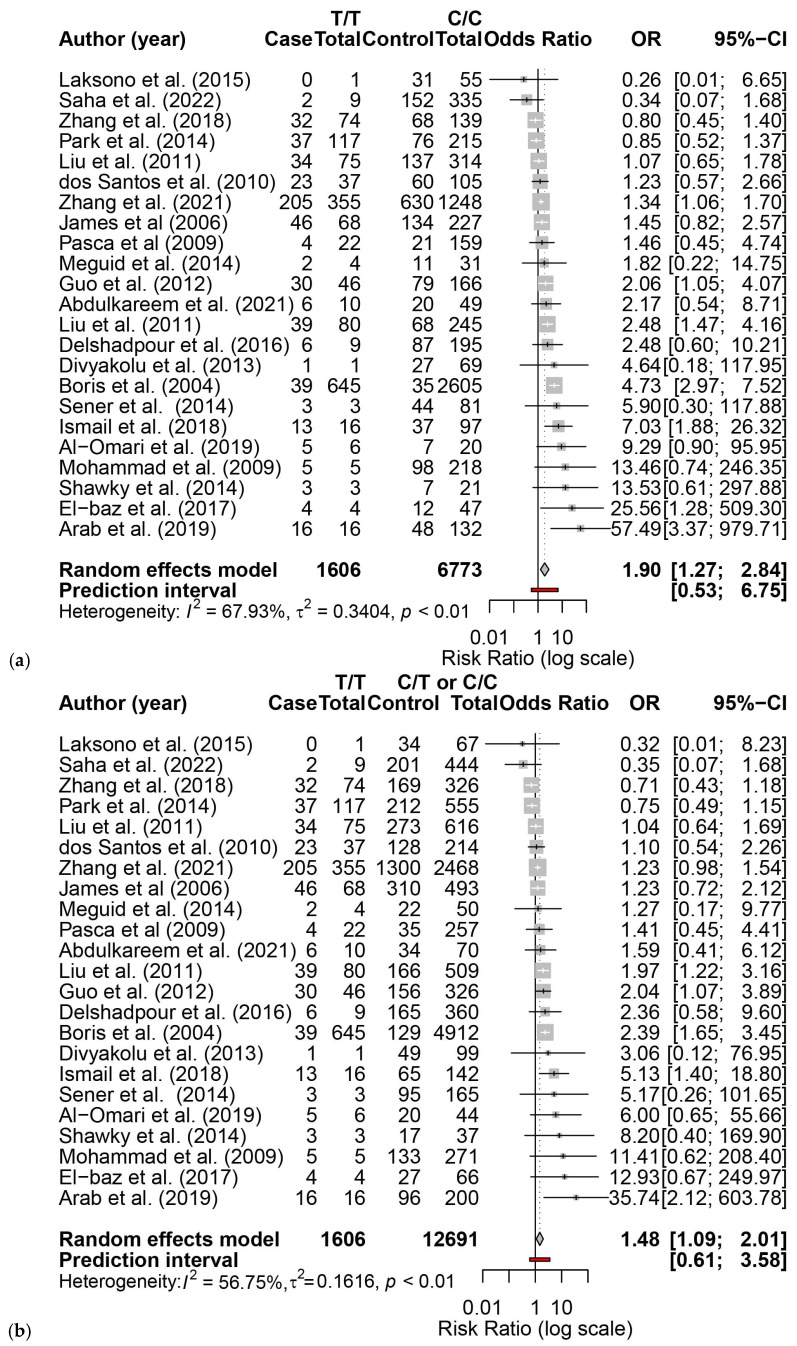
Forest plots for the pooled odds ratios of *MTHFR C677T* on ASD susceptibility. The solid vertical line represents no effect; the dashed line represents the polled effect; the diamond is centered on the pooled effect with lateral points at the 95% confidence intervals. (**a**) TT vs. CC; (**b**) TT vs. CT + CC [8,9,10,11,12,27,28,29,30,31,32,33,34,35,36,37,38,40,41,42,43,44].

**Figure 4:**
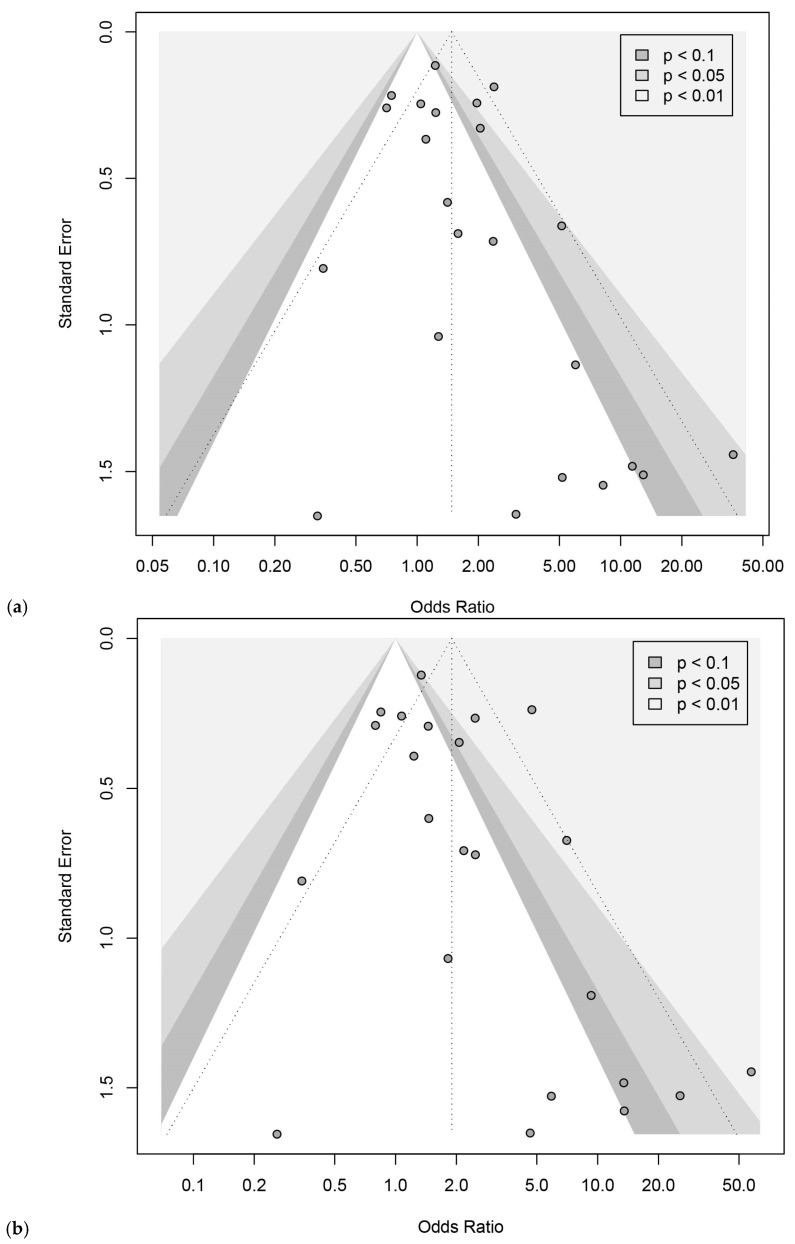
Funnel plots for the pooled odds ratios of *MTHFR C677T* on ASD susceptibility. Each dot represents an included study; the vertical dashed line represents the combined estimate of the Odds Ratio with 95% confidence interval; shaded regions distinguish significance levels (*p* < 0.1, *p* < 0.05, *p* < 0.01). (**a**) TT vs. CC; (**b**) TT vs. CT + CC.

Twenty-three articles reported the association between *MTHFR C677T* and ASD, including 13,913 subjects (4391 cases, 9522 controls) (Supplementary Table S2) [8,9,10,11,12,27,28,29,30,31,32,33,34,35,36,37,38,40,41,42,43,44]. We found that *MTHFR C677T* was significantly associated with ASD. The pooled OR of *C677T* on ASD susceptibility for a homozygous reference cohort (TT vs. CC) was 1.90 (95% CI: 1.27, 2.84) (Figure 3a), and when including heterozygosity in the control cohort (TT vs. CT + CC), it was 1.48 (95% CI: 1.09, 2.01) (Figure 3b). There was moderate to substantial heterogeneity among the studies (TT vs. CC: I^2^ = 67.9%, *p* < 0.0001; TT vs. CT + CC: I^2^ = 56.8%, *p* = 0.0004). Funnel plots for TT vs. CC showed no evidence of the small-study effect (Egger’s intercept = 0.97, t = 1.77, *p* = 0.091) (Figure 4a), while TT vs. CT + CC showed evidence of a small-study effect (Egger’s intercept = 1.80, t = 2.33, *p* < 0.03) (Figure 4b). Alternative models were explored, and they also demonstrated susceptibility and small-study effects (Supplementary Figure S1). Leave-one-out sensitivity analysis showed that exclusion of any single study did not materially change the pooled OR for TT vs. CC (Supplementary Figure S2A) and TT vs. CT + CC (Supplementary Figure S2B), indicating that the association between the *MTHFR C677T* genotype and ASD risk was robust.

**Figure 5:**
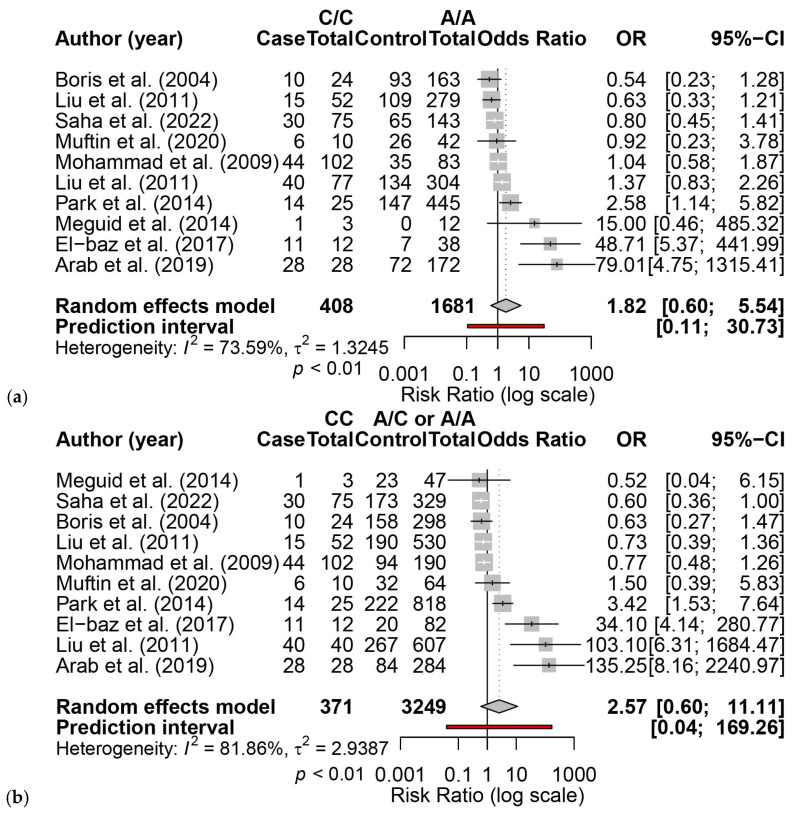
Forest plots for the pooled odds ratios of *MTHFR A1298C* on ASD susceptibility. The solid vertical line represents no effect; the dashed line represents the polled effect; the diamond is centered on the pooled effect with lateral points at the 95% confidence intervals. (**a**) CC vs. AA; (**b**) CC vs. AC + AA [9,12,28,29,36,38,39,40,42].

**Figure 6:**
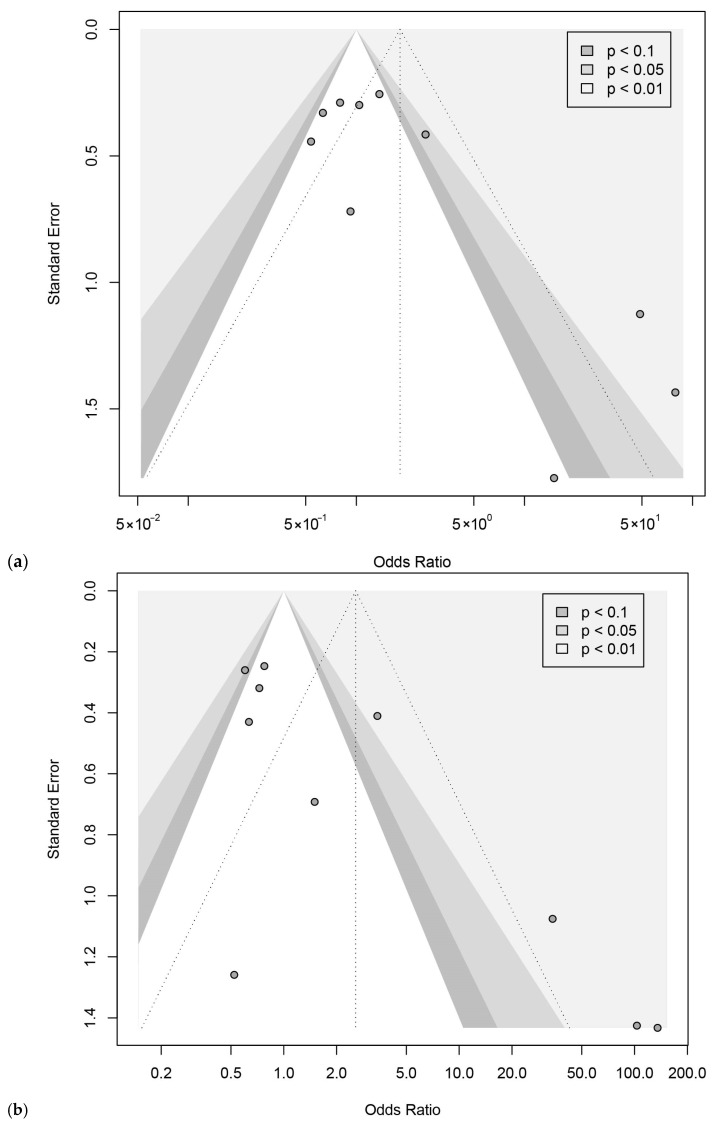
Funnel plots for the pooled odds ratios of *MTHFR A1298C* on ASD susceptibility. Each dot represents an included study; the vertical dashed line represents the combined estimate of the Odds Ratio with 95% confidence interval; shaded regions distinguish significance levels (*p* < 0.1, *p* < 0.05, *p* < 0.01). (**a**) CC vs. AA; (**b**) CC vs. AC + AA.

Ten articles, including 3009 subjects (1462 cases, 1547 controls), evaluated the link between *MTHFR A1298C* and ASD susceptibility (Supplementary Table S3) [9,12,28,29,36,38,39,40,42]. No significant association between *MTHFR A1298C* and ASD was found. The pooled OR of *A1298C* on ASD susceptibility for a homozygous reference cohort (CC vs. AA) was 1.82 (95% CI: 0.60, 5.54) (Figure 5a), and when including heterozygosity in the control cohort (CC vs. CA + AA), it was 2.57 (95% CI: 0.60, 11.11) (Figure 5b). Funnel plots for CC vs. AA (Egger’s intercept = 2.53, t = 2.41, *p* < 0.043) (Figure 6a) and CC vs. CA + AA (Egger’s intercept = 3.18, t = 3.15, *p* < 0.014) (Figure 6b) showed evidence of small-study effect. Trim-and-fill analysis altered the ORs of CC vs. AA (OR = 1.16, 95% CI: 0.25, 5.33) and CC vs. CA + AA (OR = 1.23, 95% CI: 0.18, 8.22). Alternative models did not indicate susceptibility but revealed small-study effects (Supplementary Figure S3). Leave-one-out sensitivity analysis showed that the pooled OR remained non-significant after exclusion of each individual study, indicating that the null association between CC vs. AA (Supplementary Figure S4A) and CC vs. CA + AA (Supplementary Figure S4B) was robust.

In a subgroup analysis by race/ethnicity, the African (Egyptian) cohort with *MTHFR C677T* variants had a significantly higher ASD susceptibility than Asian cohorts across all models (TT vs. CC: coefficient = −1.49, 95% CI = −2.93, −0.047, *p* = 0.044; TT vs. CT + CC: coefficient = −1.28, 95% CI = −2.55, −0.0052, *p* = 0.049; CT vs. CC: coefficient = −0.90, 95% CI = −1.73, −0.071, *p* = 0.035; CT + TT vs. CC: coefficient = −1.0192, 95% CI = −1.92, −0.12, *p* = 0.028). However, the African (Egyptian) *C677T* cohort had a significantly higher ASD susceptibility than the European cohort only in models that considered heterozygosity with the affected group (CT vs. CC: coefficient = −0.91, 95% CI = −1.77, −0.053, *p* = 0.039; CT + TT vs. CC: coefficient = −1.04, 95% CI = −1.98, −0.10, *p* = 0.031) (Table 1).

We also examine the association between the *MTHFR C677T* variant and ASD in the African (Egyptian) cohort compared to other ethnic cohorts using multiple meta-regression analyses. For each genetic model, covariates were selected based on their ability to explain the largest proportion of between-study heterogeneity (Supplementary Table S4). Specifically, study quality was adjusted for in the TT vs. CC model; publication year was adjusted for in the TT vs. CT + CC model; and both publication year and diagnostic tool were adjusted for in the CT vs. CC and CT + TT vs. CC models. The multiple meta-regression results indicated that the African (Egyptian) cohort carrying *MTHFR C677T* variants had a significantly higher ASD susceptibility than Asian cohorts in TT vs. CC (coefficient = −1.58, 95% CI = −3.04, −0.11, *p* = 0.036) but not in the other genotypes (Supplementary Table S5).

Similarly, the African (Egyptian) cohort with *MTHFR A1298C* variants also had a significantly higher ASD susceptibility than the Asian and European cohorts in models that considered heterozygosity with the affected group (Table 2). Compared to the Asian cohort, the heterozygous models reached significance, and the homozygous model approached significance (AC vs. AA: coefficient = −1.91, 95% CI = −3.47, −0.35, *p* = 0.023; AC + CC vs. AA: coefficient = −2.18, 95% CI = −4.25, −0.12, *p* = 0.041; CC vs. AA: coefficient = −3.18, 95% CI = −6.36, 0.0091, *p* = 0.05). Compared to the European cohort, the homozygous model also reached significance (CC vs. AA: coefficient = −3.73, 95% CI = −6.94, −0.51, *p* = 0.029; AC vs. AA: coefficient = −2.60, 95% CI = −4.16, −1.04, *p* = 0.0056; AC + CC vs. AA: coefficient = −3.036, 95% CI = −5.16, −0.91, *p* = 0.012).

Using the same approach as for the *MTHFR C677T* variant, we conducted multiple meta-regression analyses to examine the association between the *MTHFR A1298C* variant and ASD in the African (Egyptian) cohort compared with other ethnic cohorts (Supplementary Table S6). Specifically, the diagnostic tool was adjusted for in the CC vs. AA, CC vs. AC + AA, and AC + CC vs. AA models, whereas publication year and study quality were adjusted for in the AC vs. AA model. In the results, the heterozygous model AC vs. AA retained higher ASD risk in the African (Egyptian) compared with the Asian cohort (coefficient = −1.83, 95% CI = −3.60, −0.06, *p* = 0.045) and the European cohort (coefficient = −2.56, 95% CI = −4.54, −0.59, *p* = 0.021), whereas no significant associations were observed for other genotype models (Supplementary Table S7).

## 4. Discussion

In this meta-analysis, we revisited the *MTHFR* gene variants with a reported association with ASD—*C677T* and *A1298C*—including additional studies that expand coverage of racial/ethnic cohorts used for assessing this association. Our study is consistent with previous and current reports that support the association between *MTHFR C677T* and ASD susceptibility, as well as the lack of association between *MTHFR A1298C* and ASD [45]. However, these associations maintained high residual heterogeneity, and further investigation of the study population structure via a meta-regression analysis revealed higher associations with ASD for the African (Egyptian) cohort than other ethnic cohorts. Interestingly, the comparison between the African (Egyptian) and European cohorts for *C677T* returned significance only in models treating heterozygosity as an affected group, suggesting a role for haploinsufficiency in the African (Egyptian) subgroup. Similar findings were found for *A1298C*; though this allele did not meet statistical significance as an ASD risk factor in our analysis, differential contributions of heterozygosity between subgroups could provide a biological basis contributing to the heterogeneity in the model.

The observed increase in *MTHFR* variant risk for ASD in the African (Egyptian) cohort should be interpreted with caution. It is notable that the African (Egyptian) cohort is less well-represented compared to Asian and European cohorts in both the number of independent studies (for *C677T*, 4 vs. 12 and 7, respectively) and cohort size (for *C677T*, 153 vs. 2914 and 1324, respectively). Considering that studies including African ancestry and meeting criteria represented a specifically Egyptian population, these results may not be generalizable to the larger African continent. We anticipate that ongoing projects like the Egyptian Genome Project will expand the available genetic information and diagnostics within this cohort [46].

With a larger cohort membership, it is expected that the allele frequency estimate for the *MTHFR* variant will improve. Data from the Egyptian Genome Project [47] provides allele frequencies for C677T and A1298C at 29.5% and 30.7%, respectively, similar to the reported frequencies for other populations in gnomAD (C677T ~34% in European and East Asian and A1298C = 31% in European); however, gnomAD frequencies for both alleles in African/African American ancestry groups are notably lower (~10% and 16%, respectively) [48]. At the same time, similar additional studies are expected to narrow the ASD prevalence estimate in Africa, which varies widely from 0.33% to 33.6% [49], whereas global estimates are ~0.8% (1 in 127 people) [50]. If future expansion of African cohorts results in values approaching those of the other cohorts, the differentiation among these populations in our study may disappear. However, for other conditions (myocardial infarction and retinal venous ocular disease), the *MTHFR C677T* has been specifically reported to confer differential risk by “racial” group (European vs. other), raising the possibility that suggested differences in allele susceptibility may remain even as cohort representation improves [51]. Broadly speaking, other African subpopulations may reasonably return unique results, especially given the vastly increased genetic diversity recently observed in South Africa, the most geographically distant region from our cohort [52].

However, critical differences in genetic and social contributions to ASD susceptibility that cut across racial/ethnic boundaries would counter convergence, and the relatively lower frequency of these *MTHFR* variants may reflect genetically modified increased susceptibility in African cohorts. Other genetic architectures for *MTHFR* are also being revealed by additional sequencing. For example, *T1317C* is a more common *MTHFR* variant in African (African American and South African) than Asian (Han and Uyghur) populations or North American cohorts (35%, ~11%, and ~5%, respectively) [47]. Even *MTHFR* variants with positive associations, such as the “anti-hypertensive” intronic rs17367504 variant, have been reported in Egyptian cohorts with similar frequency to European cohorts (AF = 0.145 vs. AF = 0.139, respectively), although with fewer proxy variant associations, reflective of a shorter haplotype block and distinct variant combinations in the Egyptian *MTHFR* structure [47]. Whether *MTHFR* genetic architectures, including *T1317C* or rs17367504, have intragenic interactions with *C677T* or *A1298C* or contribute to ASD susceptibility risk is currently unknown. Whether the risk factors identified in this work for an African (Egyptian) cohort are extensible to other African genetic architectures for *MTHFR* is also currently unknown.

The social contributions to ASD prevalence complicate attribution to genetic causes. The United States (US) provides an example where the overall ASD prevalence remains at ~1% despite observing more individuals—a result driven by an explosion in ASD diagnoses (~2.7%) among children and young adults (25–34 years old) that may skew future rates even higher. Not only is ASD prevalence high in the US, but it is higher still among White US Medicaid enrollees; curiously, *MTHFR C677T* and *A1298C* variants are underrepresented in this population. However, among a cohort of 7 million English students, Black pupils had the highest standardized prevalence (though *MTHFR* variant status is unknown) [53]. In both cases, socioeconomic disadvantages play a moderating role that cannot be ignored. In resource-limited environments, ASD diagnostics become even more challenging, with social stigma and poor medical access contributing to late diagnosis or underdiagnosis [54]. Few studies have been conducted within these environments, but ascertainment bias and a lack of standardized phenotyping have been proposed as explanations for more severe ASD in African populations [55]. Our work presents a biological plausibility, and we agree that technological advancements could assist with disentangling the effects of socioeconomic barriers from biological causality.

Technological advancements in machine learning and artificial intelligence can assist in connecting genotype and phenotype through physiological measures, making the most of the limited datasets that measure intermediate physiology like transcriptomes, plasma homocysteine, or motor deficits. These tools can also be used to assist with the collection of more granular phenotypic or behavioral measurements through ambient listening and automatic language translation in clinics. Inexpensive wearable technology can even generate continuous measures for analysis by machine learning without requiring expert knowledge, invasive procedures, or excessive doctor visits from the user [56]. Despite the excitement and promise, implementations in resource-limited settings remain a future objective, as even the most inexpensive technology and streamlined tools still require a financial investment and a networked infrastructure at a minimum.

Ultimately, understanding the contributions of known, associated common genetic variation in the phenotypic outcomes of disorders like ASD will allow us to differentiate the causal role of these variants and assign appropriate interventions among individuals based on their characteristic genotypes. Access to healthcare, affordable technology, and inexpensive treatment options will all be critical for improving health globally for those with ASD.

## Figures and Tables

**Table 1:** Meta-regression comparing the association between the *MTHFR C677T* variant and ASD in the African/Egyptian cohort compared to other ethnic cohorts.

C677T (Ref: African/Egyptian)	Coefficient (95% CI)	*p* Value ^1^
TT vs. CC		
**Asian**	**−1.4898 (−2.9326, −0.0469)**	**0.0436**
European	−1.2200 (−2.6713, 0.2313)	0.0948
TT vs. CT + CC		
**Asian**	**−1.2757 (−2.5463, −0.0052)**	**0.0492**
European	−1.0353 (−2.3077, 0.2371)	0.1051
CT vs. CC		
**Asian**	**−0.9011 (−1.7315, −0.0708)**	**0.0349**
**European**	**−0.9102 (−1.7677, −0.0528)**	**0.0386**
CT + TT vs. CC		
**Asian**	**−1.0192 (−1.9194, −0.1191)**	**0.0284**
**European**	**−1.0403 (−1.9775, −0.1030)**	**0.0313**

^1^ Rows with *p* < 0.05 are bolded.

**Table 2:** Meta-regression comparing the association between the *MTHFR A1298C* variant and ASD in the African/Egyptian cohort compared to other ethnic cohorts.

A1298C (Ref: African/Egyptian)	Coefficient (95% CI)	*p* Value ^1^
CC vs. AA		
Asian	−3.1774 (−6.3638, 0.0091)	0.0505
**European**	**−3.7256 (−6.9428, −0.5084)**	**0.0290**
CC vs. AC + AA		
Asian	−0.6144 (−2.6947, 5.7357)	0.4219
European	−0.6147 (−5.9095, 4.6801)	0.7916
AC vs. AA		
**Asian**	**−1.9119 (−3.4734, −0.3505)**	**0.0231**
**European**	**−2.6020 (−4.1620, −1.0420)**	**0.0056**
AC + CC vs. AA		
**Asian**	**−2.1850 (−4.2526, −0.1175)**	**0.0411**
**European**	**−3.0362 (−5.1581, −0.9142)**	**0.0117**

^1^ Rows with *p* < 0.05 are bolded.

## Data Availability

The original contributions presented in the study are included in the article/Supplementary Material, further inquiries can be directed to the corresponding author.

## Supplementary Materials

The following supporting information can be downloaded at https://www.mdpi.com/article/10.3390/brainsci16010093/s1, Figure S1: Forest plot for C677T heterozygous and haploinsufficient models; Figure S2: (A) Forest plot for leave-one-out sensitivity analyses for C677T TT vs. CC. (B) Forest plot for leave-one-out sensitivity analyses for C677T TT vs. CC + CT; Figure S3: Forest plot for A1298C heterozygous and haploinsufficient models; Figure S4: (A) Forest plot for leave-one-out sensitivity analyses for A1298C CC vs. AA. (B) Forest plot for leave-one-out sensitivity analyses for A1298C CC vs. CA + AA; Table S1: Newcastle–Ottawa Scale Star score; Table S2: Articles and cohort numbers available for assessing the C677T variant; Table S3: Articles and cohort numbers available for assessing the A1298C variant; Table S4: Comparison of meta-regression models examining the association between the *MTHFR C677T* variant and ASD in African versus non-African populations; Table S5: Multiple meta-regression comparing the association between the *MTHFR C677T* variant and ASD in the African cohort compared to other ethnic cohorts; Table S6: Comparison of meta-regression models examining the association between the *MTHFR A1298C* variant and ASD in African versus non-African populations; Table S7: Multiple meta-regression comparing the association between the *MTHFR A1298C* variant and ASD in the African cohort compared to other ethnic cohorts. Supplemental File: PRISMA 202 checklist [57].

## Abbreviations

The following abbreviations are used in this manuscript:

ASD	Autism Spectrum Disorder
MTHFR	5,10-methylenetetrahydrofolate reductase

## Appendix A

The PRISMA 2020 checklist is provided separately as a Supplemental File. Search strategy and screening per database are provided.

Search strategy: Original human studies; publication date: 1980–present (6 August 2023); English language; databases: PubMed, Medline, PsycINFO; form of results: pooled effect size for (1) odds ratio representing the association of MTHFR variants between ASD and control groups and (2) Cohen’s d/Hedges’ g representing the difference between the means of metabolites in ASD and control groups. Search query, default setting: MTHFR and ASD: ((MTHFR) OR (methylenetetrahydrofolate reductase) OR (677C-T) OR (C677T) OR (677C>T) OR (1298CC) OR (1298A>T) OR (1298AC) OR (A1298C)) AND ((Autism) OR (ASD) OR (Autism Spectrum Disorders)).

**Table A1:** Search results and exclusion reported per database.

	PubMed	Medline	PsycINFO
Total results	73	60	4
Exclusions (reason)	6 (no alleles) 13 (non-ASD) 9 (non-human) 2 (no odds ratio) 4 (case report) 1 (non-English)	59 (no alleles)	4 (no alleles)
