# Supplementary figures and images for: The Contribution of Ethnicity to the Association of MTHFR Variants C677T and A1298C with Autism Spectrum Disorder: A Meta-Analysis
Source: Brain Sci. 2026 Jan 16;16(1):93. doi: 10.3390/brainsci16010093 (PMC12838728; doi:10.3390/brainsci16010093)

Supplementary Figure S1A

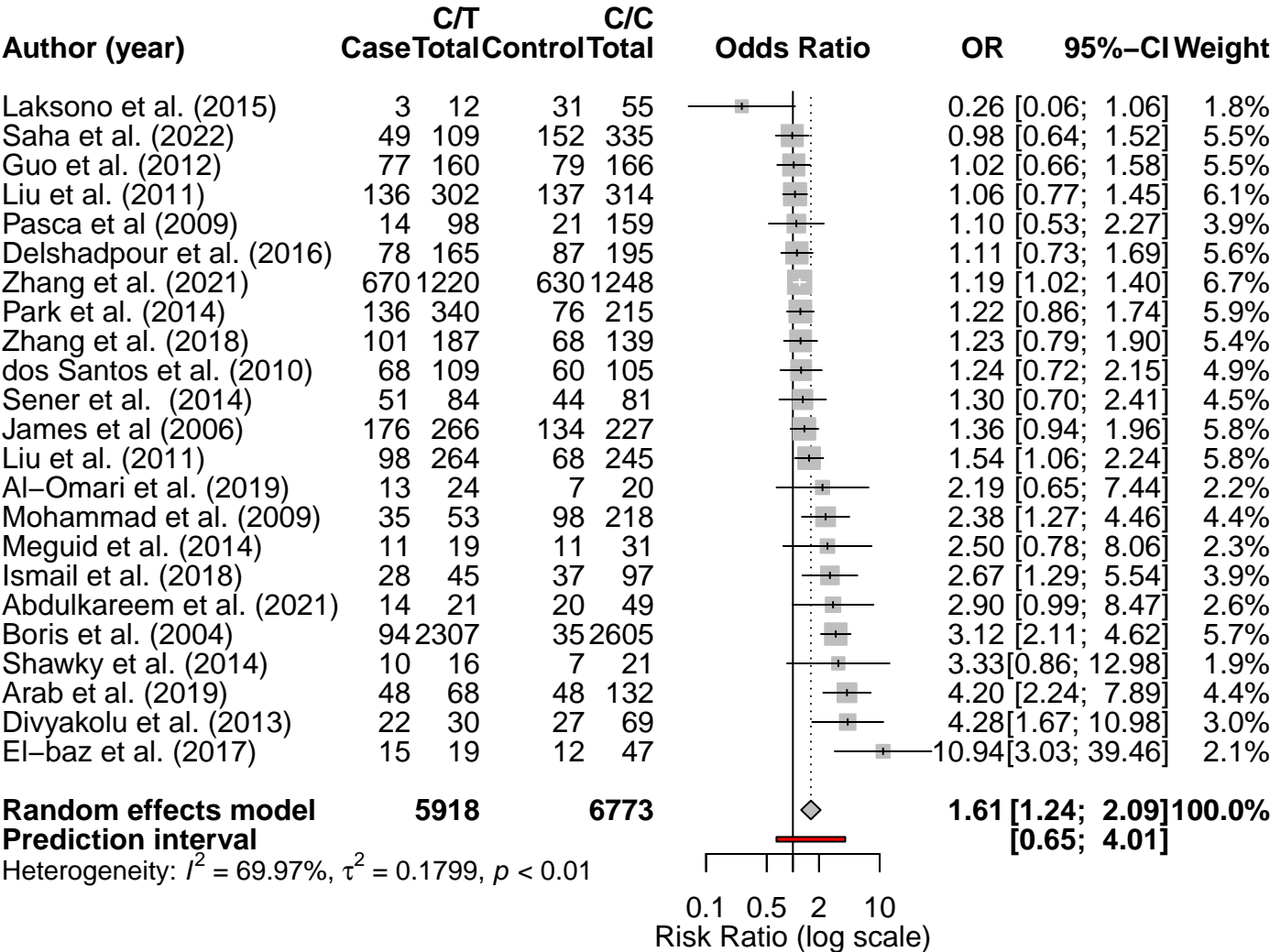

Supplement: Supplementary file 1 [file brainsci-16-00093-s001.zip › Figure S1A_forest_677-HET.pdf]

Supplementary Figure S1B

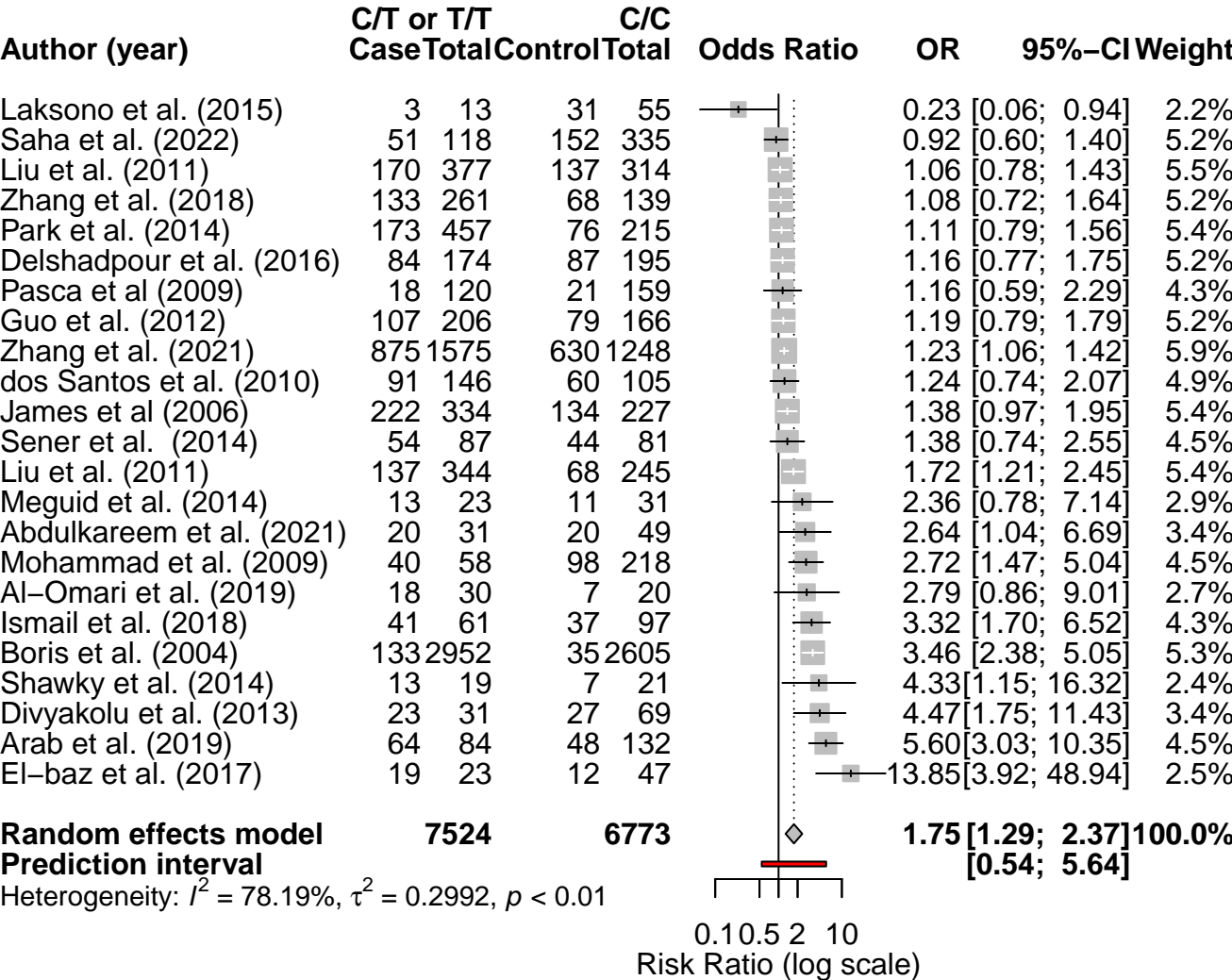

Supplement: Supplementary file 1 [file brainsci-16-00093-s001.zip › Figure S1B_forest_677-HAPLO.pdf]

Supplementary Figure S2A

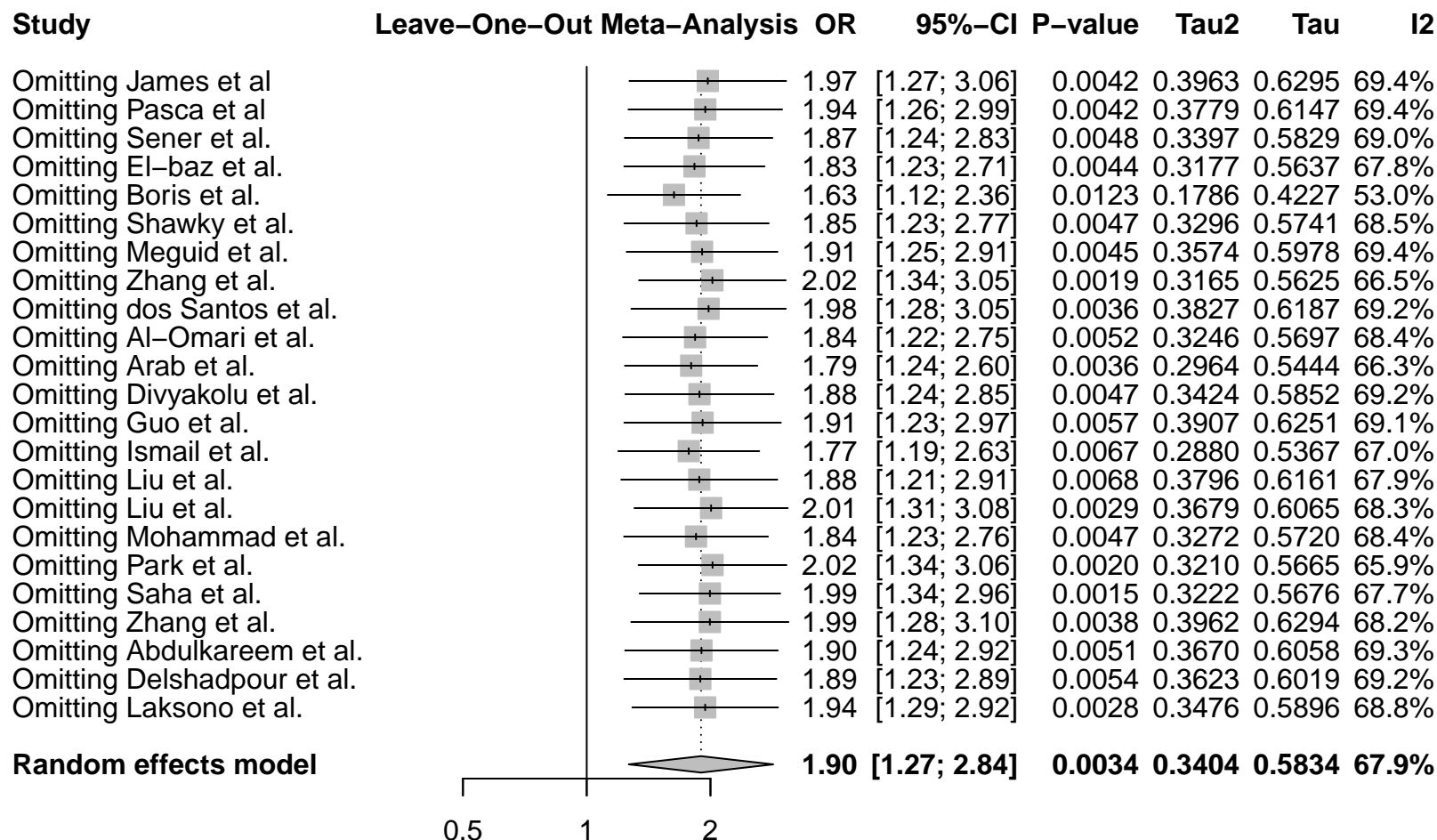

Supplement: Supplementary file 1 [file brainsci-16-00093-s001.zip › Figure S2A_677SA.pdf]

Supplementary Figure S2B

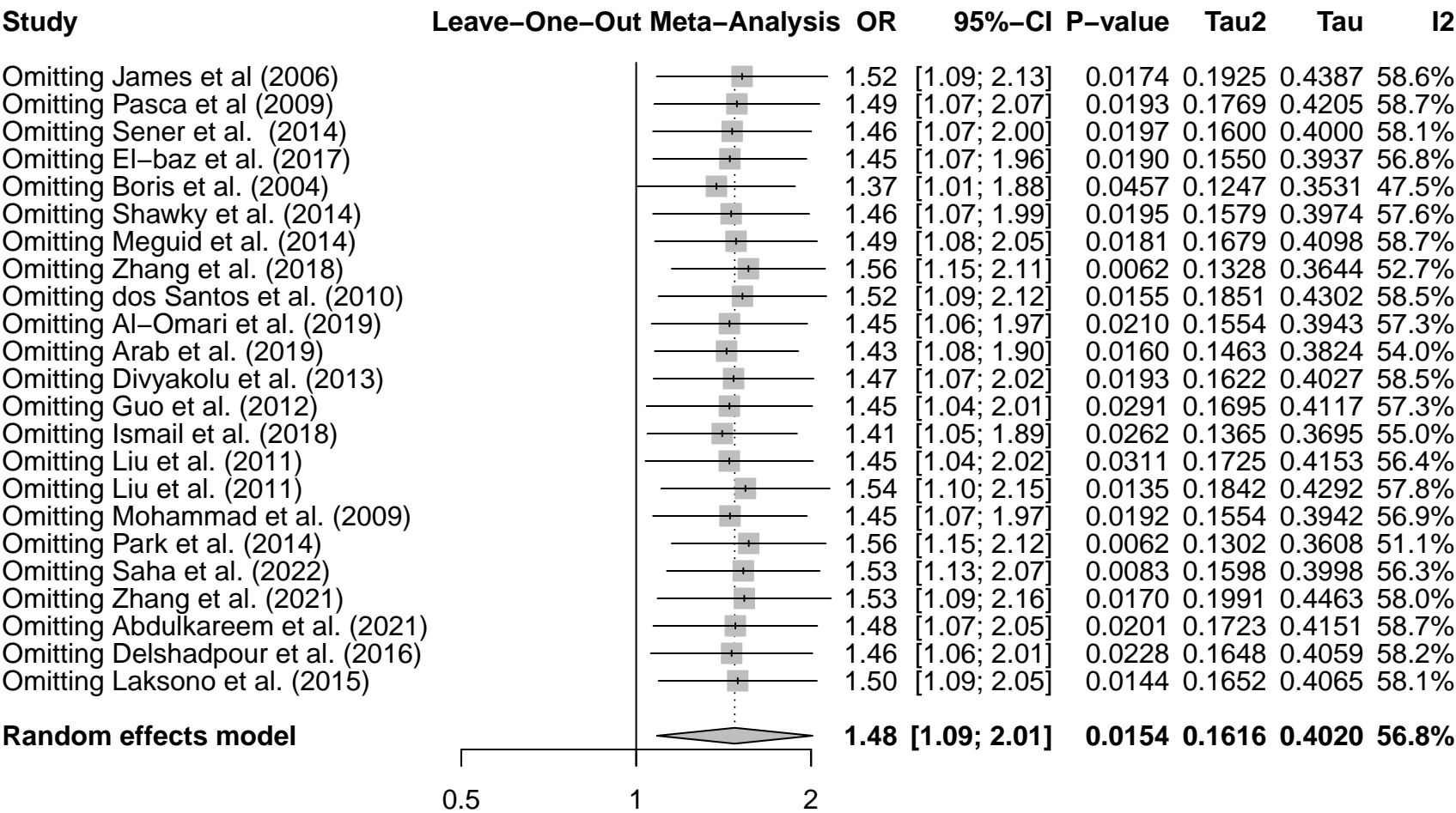

Supplement: Supplementary file 1 [file brainsci-16-00093-s001.zip › Figure S2B_677SA.pdf]

Supplementary Figure S3A

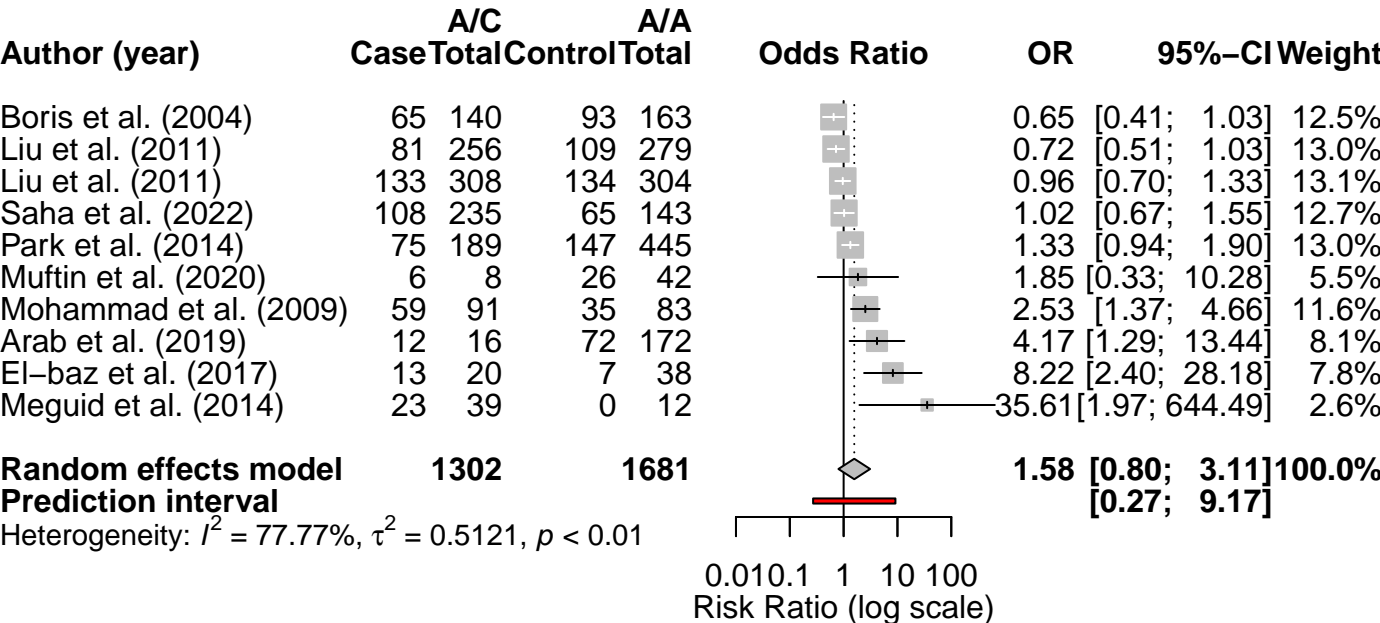

Supplement: Supplementary file 1 [file brainsci-16-00093-s001.zip › Figure S3A_forest_1298-HET.pdf]

Supplementary Figure S3B

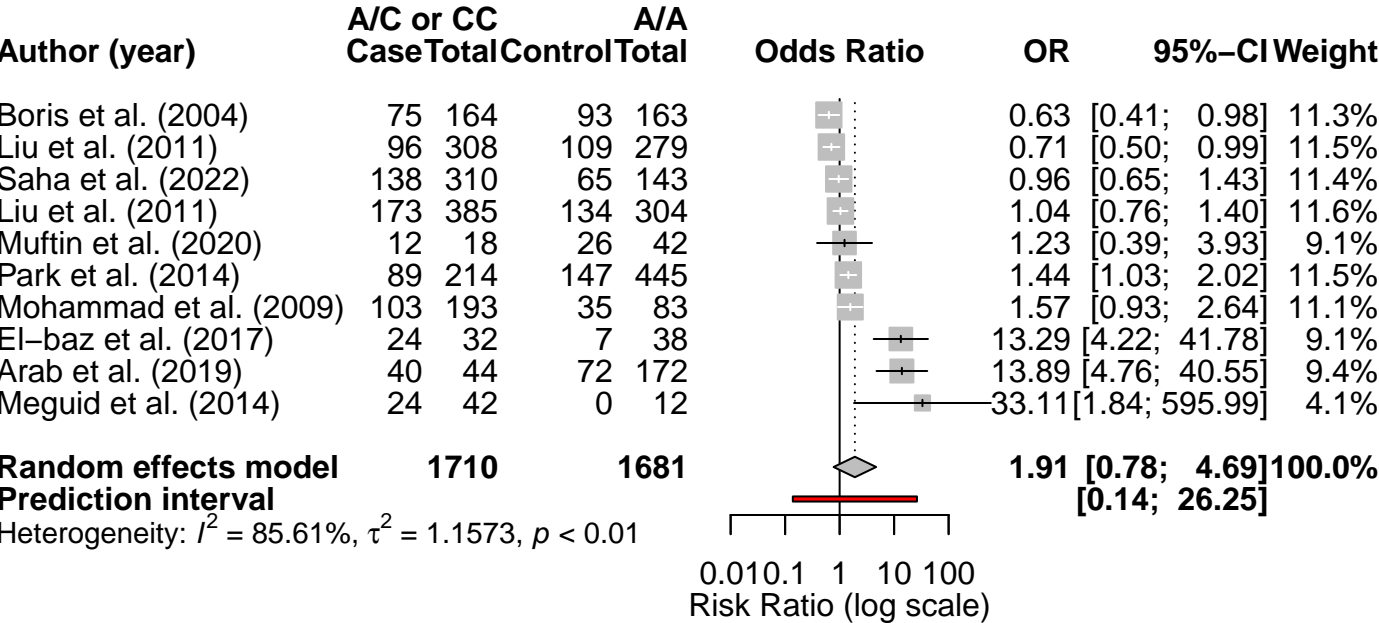

Supplement: Supplementary file 1 [file brainsci-16-00093-s001.zip › Figure S3B_forest_1298-HAPLO.pdf]

Supplementary Figure S4A

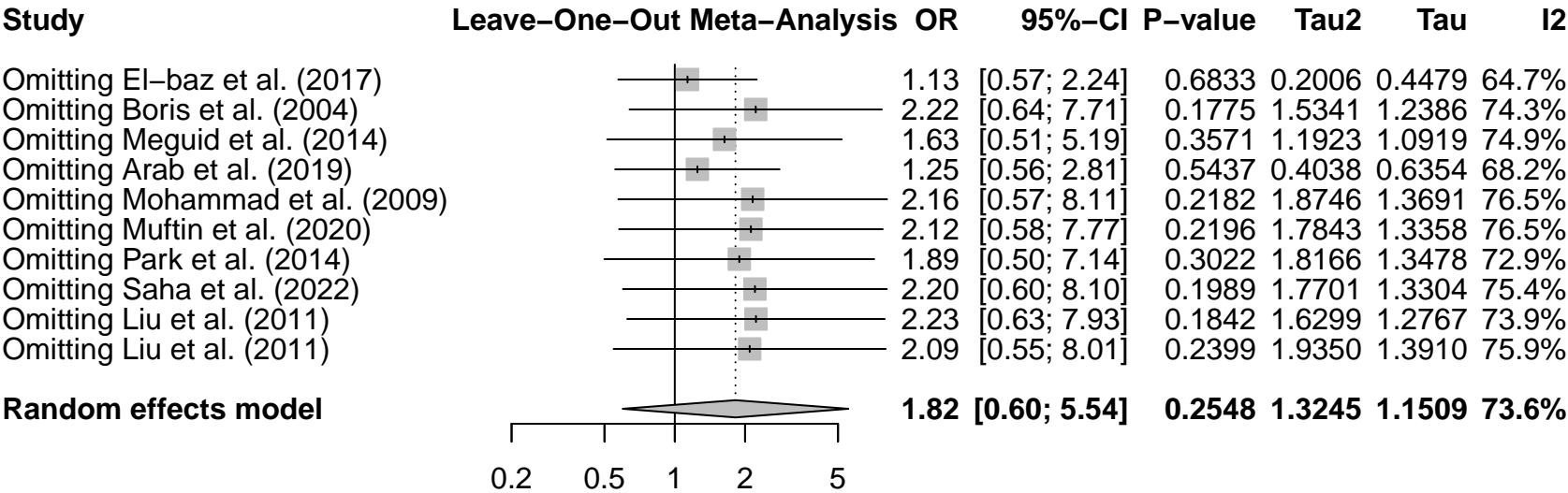

Supplement: Supplementary file 1 [file brainsci-16-00093-s001.zip › Figure S4A_1298SA.pdf]

Supplementary Figure S4B

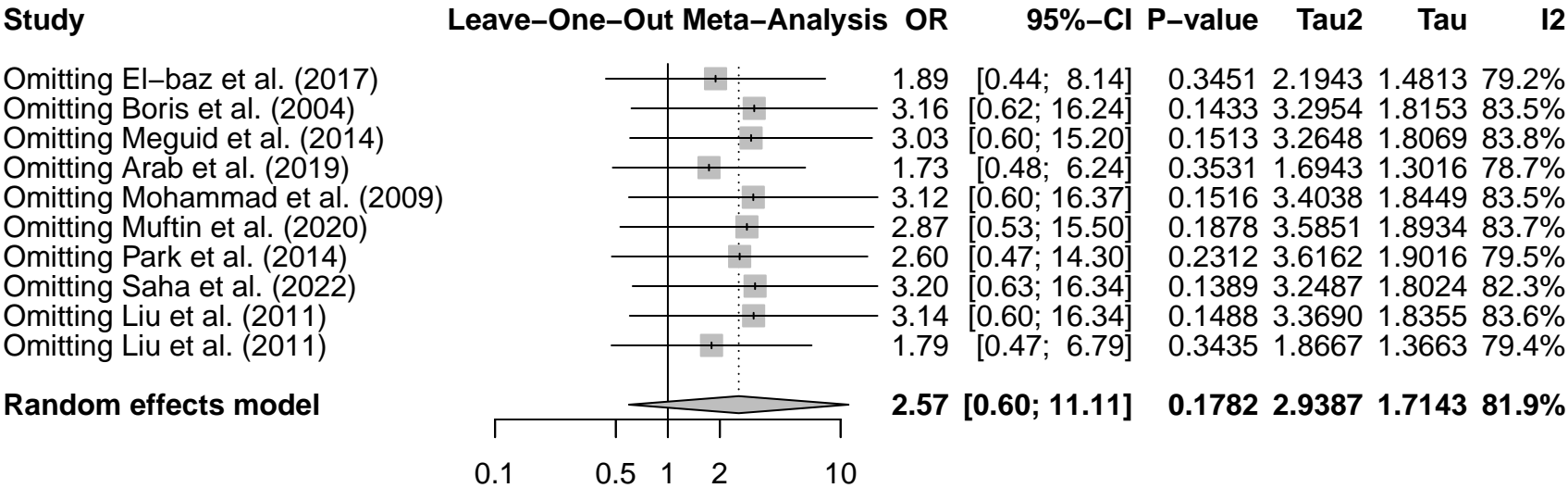

Supplement: Supplementary file 1 [file brainsci-16-00093-s001.zip › Figure S4B_1298SA.pdf]
